# A randomized, double-blind, placebo-controlled and dose-ranging study to evaluate the safety and efficacy of XG005 in subjects with painful osteoarthritis of the knee

**DOI:** 10.1016/j.ocarto.2025.100737

**Published:** 2025-12-19

**Authors:** Limin Ren, Wenjie Zheng, Zhenpeng Guan, Yang Zhang, Zeyu Huang, Tong Li, Yuwei Peng, Qiuli Wu, Wei Gou, Wei Zhao, Pengyan Qiao, Xiaoli Pan, Guang-Liang Jiang

**Affiliations:** a Peking University, People's Hospital, Rheumatology and Immunology Department, Beijing, China; b Yantai Yuhuangding Hospital of Qingdao University, Department of Orthopedics, Yantai, China; c Peking University, Shougang Hospital, Department of Orthopedics, Beijing, China; d Division of Orthopaedic Surgery, Nanfang Hospital, Southern Medical University, Guangzhou, China; e West China Hospital of Sichuan University, Department of Orthopedics, Chengdu, China; f Xiangya Hospital of Central South University, Rheumatology and Immunology Department, Changsha, China; g PingXiang People's Hospital, Rheumatology and Immunology Department, Pingxiang, China; h Tianjin Medical University General Hospital, Department of Orthopedics, Tianjin, China; i Hebei PetroChina Central Hospital, Rheumatology and Immunology Department, Langfang, China; j Central Hospital Affiliated to Shenyang Medical College, Department of Orthopedics, Shenyang, China; k Shanxi Bethune Hospital, Shanxi Academy of Medical Sciences, Third Hospital of Shanxi Medical University, Tongji Shanxi Hospital, Rheumatology and Immunology Department, Taiyuan, China; l Affiliated Hospital of Zunyi Medical University, Rheumatology and Immunology Department, Zunyi, China; m Xgene Pharmaceutical, Inc, China

**Keywords:** XG005, Osteoarthritis, Pain, Symptoms, Naproxen, Pregabalin

## Abstract

**Objective:**

Symptomatic treatment for osteoarthritis (OA) remains a major unmet need. This trial evaluated XG005, a novel, non-opioid, dual-mechanism agent targeting both inflammatory and neuropathic pathways in patients with knee OA.

**Design:**

This randomized, double-blind, placebo-controlled study enrolled 318 patients with moderate-to-severe knee OA pain to receive XG005 750 mg, 500 mg, or placebo twice daily for 4 weeks. Efficacy measures included weekly average of daily walking pain (WADWP), Western Ontario and McMasters Universities Osteoarthritis Index (WOMAC), Knee Injury and Osteoarthritis Outcome Score (KOOS), Patient Global Impression of Change (PGIC), Sleep Interference Score (SIS), and Short-Form-12 Health Survey (SF-12).

**Results:**

The primary efficacy endpoint of improvement from baseline in WADWP at week 4 was statistically greater for the 750 mg group than placebo with least squares mean difference (LSMD) (95 % CI) of −0.55 (−0.94, −0.16). Key secondary endpoints were significantly improved for high- and low-dose XG005 compared to placebo at week 4, with LSMD (95 % CI) of −0.43 (−0.74, −0.13) and −0.4 (−0.78, −0.02) in WOMAC pain, and 5.56 (2.69, 8.42) and 3.74 (0.17, 7.31) in KOOS pain. WOMAC and KOOS stiffness and function, PGIC, SIS and SF-12 mental and general health showed statistically significant improvements over placebo. Patients with neuropathic pain had approximately 2-3-fold greater symptom improvements than patients with nociceptive pain. Mild dizziness and somnolence were most seen in XG005 groups.

**Conclusion:**

XG005 demonstrated consistent efficacy in improving OA symptoms with acceptable tolerability. Additional studies in OA patients with neuropathic pain are needed to confirm its dual mechanism advantages.

**Clinical trial registration:**

CTR20222406.

## 1 Introduction

In the absence of approved therapies that alter the structural progression of osteoarthritis (OA), treatment options remain largely symptomatic with analgesics as a foundation of OA management [1,2]. The existing analgesics, such as non-steroidal anti-inflammatory drugs (NSAIDs) or intra-articular corticosteroids, mainly act on inflammatory pain and are often associated with limited efficacy and/or safety concerns [3,4]. Opioids, though sometimes used, pose serious risks of dependence and adverse effects [3]. In chronic OA, the challenges arise in part from the development of neuropathic pain mechanisms on top of the underlying inflammatory pathogenesis, which create complex pain phenotypes that explain why OA pain can be challenging to treat effectively [[5], [6], [7], [8]].

An analgesic effective for both nociceptive and neuropathic pain could provide meaningful clinical benefits and address the limitations of current standard-of-care therapies, even in the absence of structural modification.

XG005 is a novel, non-opioid, small-molecule new chemical entity created via conjugating naproxen and pregabalin to synchronize their actions. Thus, it has a dual mechanism of actions: inhibition of cyclooxygenase (COX) enzymes involved in inflammatory pain and modulation of calcium channel subunits implicated in neuropathic pain signaling. Recently, XG005 demonstrated potent analgesic effects in acute post-bunionectomy pain (*Cohen's d* = 1.55 over placebo), a model with the mixture of nociceptive and neuropathic pain [9]. It remained unclear whether XG005 could provide strong analgesia and improve symptoms of joint stiffness and dysfunction in chronic knee OA, a condition with nociceptive and/or neuropathic components.

In this pilot trial of XG005 in osteoarthritis, we assessed its efficacy and safety in patients with knee OA and compared its effects on nociceptive versus neuropathic pain to inform future patient selection strategies.

## 2 Methods

### 2.1 Study design and study subjects

This proof-of-concept trial was a 3-arm, double-blind, placebo-controlled, parallel-group, multiple-center, randomized study to characterize the analgesic effect and safety for two dose levels (750 mg and 500 mg) of XG005 versus placebo twice daily (BID) in patients with painful OA of the knee at 23 hospitals in China (CTR20222406). It consisted of a screening period (up to 4 weeks), randomization, dose titration (third day to reach 500 mg BID and 7th day to reach 750 mg BID) and stable dose treatment for 4 weeks, follow-up visits at week 1, 2, and 4, and phone safety follow-up one week after dosing completion [[10], [11], [12]]. 4 weeks were chosen as the treatment duration in this pilot study because naproxen is one of the active metabolites of XG005. And Asians are particularly sensitive to NSAIDs-induced gastrointestinal side effects and treatment guidelines recommend using the lowest possible dose and shortest duration (as short as one month even efficacious) [13,14]. Dosing duration in future studies would be justified based on the safety data from this trial. During the screening period, if eligible subjects were on pain medication or other pain therapies they were required to go through the washout process [12]. The duration for pain medication washout was at least 5 half-lives of the medication. Other pain therapies (e.g., physical therapies) were stopped at least 2 days prior to starting baseline recording.

Qualified subjects recorded OA pain intensity for the study knee while walking on a flat surface during the last 24 h, using an 11-point numeric rating scale (0–10 NRS, 0 = no pain, 10 = worst pain possible) on the eDiary, for the baseline (day −7 to −1) and 4 weeks treatment period. The baseline average knee pain was required to be at least 5.0 NRS based on at least 5 records. For bilateral knee OA, the most painful knee was designated as the study knee or arbitrarily chosen when both knees had the same baseline pain score. Pain at sites other than the study knee (if present) was required to be ≤ 3.0 [[10], [11]].

During the washout period and throughout the study, subjects were allowed to take acetaminophen as rescue pain treatment as needed [10,12] but not exceeding 2000 mg per day for up to 10 days of consecutive use at a time.

Subjects of 40–70 years old with primary OA of the knee for at least 6 months, diagnosed with American College of Rheumatology Clinical and Radiographic criteria [15] and Kellgren and Lawrence (KL) grade 2 and 3, were enrolled [12]. Subjects with secondary OA or joint involvement from other systemic diseases were excluded. Antidepressants and intra-articular corticosteroids and hyaluronic acids were prohibited [10,12]. PainDETECT questionnaires were used to characterize if a subject's OA pain had neuropathic pain components on day 1 [11]. Subjects with gastrointestinal (GI) diseases or recent surgeries of GI tract, or allergic history to NSAIDs were not included. Women of childbearing potential were not allowed to get pregnant or lactating during the study and 6 months after discontinuation from the study. Male subjects were required to contracept during the study and 6 months after discontinuation from the study. Subjects with severe depression or anxiety as assessed with Patient Health Questionnaire-9 (PHQ-9) and General Anxiety Disorder (GAD-7) were excluded [10]. Medication compliance was assessed by tablet counts at each visit. Rescue medication use was recorded on the eDiary. All patients were required to maintain their routine lifestyle and with no changes in activities throughout the study period [12].

### 2.2 Randomization and blinding

Subjects who met all inclusion criteria and none of the exclusion criteria were randomized (2:1:2) to either 750 mg XG005, 500 mg XG005 or placebo group. Study medications were provided as identically appearing and equal number of oral tablets, taken every morning and evening (i.e., every 12 h). An independent biostatistician generated and maintained randomization numbers and provided an independent drug packing unit for drug kit preparation and shipping. Research staff logged into an Interactive Web Response Systems (IWRS) with their specific username and password to acquire the randomization number and corresponding kit number for each enroller. The biostatistician was reachable to unblind a participant for safety reasons, if necessary, but was otherwise not involved in any other study activities. Participants and research staff responsible for assessments, monitoring, data management, and/or drafting statistical analysis plan were unaware of the participants' treatment allocation throughout the trial [10,12].

### 2.3 Assessments

Daily average pain when walking on a flat surface, rescue medication use and sleep interference score (SIS) (0–10 NRS scale with 0 being no interference and 10 being the worst possible) were recorded daily on an eDiary in a smart phone. Change from baseline of the weekly average of daily walking pain (WADWP) for 750 mg versus placebo at week 4 was the primary efficacy endpoint. Study joint pain when walking on a flat surface is the first question of Western Ontario and McMasters Universities Osteoarthritis Index 3.1 (WOMAC). Daily assessment of this pain via ediary was taken as the primary efficacy measure to avoid recall bias and reduce reporting burden compared with daily assessment of all pain questions of WOMAC. WOMAC, Knee Injury and Osteoarthritis Outcome Score (KOOS), Patient Global Impression of Change (PGIC), and Short-Form-12 Health Survey (SF-12) measures were assessed at various time points before and/or post treatment [10,12]. Key secondary efficacy endpoints included changes from baseline in WOMAC pain and KOOS pain subscale scores of the study knee at Week 4.

Laboratory, electrocardiogram (ECG) and physical examinations were conducted before treatment and at the exit visit. Treatment-emergent adverse events (TEAEs) were recorded and followed up throughout the study period or till resolution.

### 2.4 Statistical analysis

Sample size was determined based on change from baseline of high dose XG005 (i.e., 750 mg) vs. placebo in the weekly average of walking pain of the study knee at Week 4 with two sample *t*-test. With an estimated standard effect size of 0.374 over placebo (i.e., change from baseline for placebo = −4.3, estimated treatment effect = −1.72, and SD = 4.6), based on randomized, placebo-controlled NSAIDs trials in Asian knee OA subjects and other population [[16], [17], [18]], with 80 % power, at a two-tailed α = 0.05, 114 subjects per arm were needed to demonstrate superiority over placebo. To accommodate potential early terminations, the recruitment goal was approximately 125 subjects per arm for high dose and placebo arms and 63 subjects for the low dose (i.e., 500 mg) arm.

A gatekeeping strategy was employed in the statistical analysis plan (SAP) for multiplicity adjustment in this study (changed from the protocol). The primary efficacy endpoint and key secondary endpoints were sequentially tested. Each step was conducted only if the previous hypothesis test reached statistical significance. At each step, the full alpha level (α = 0.05) was consumed.

An analysis of covariance (ANCOVA) model was used, with treatment as a fixed effect, gender as a categorical variable, and baseline pain score as a covariate. The model-estimated mean differences (95 % CI) and P values for comparisons of the 750 mg and 500 mg groups versus the placebo group were given. Pain reports on the same day of rescue medication use were not calculated for the primary efficacy analysis, instead, the last observation prior to rescue medication use was carried forward (LOCF) [10,12]. The primary analysis population was modified intention-to-treat (mITT) subjects including all randomized and with at least one treatment. Sensitivity analyses for primary efficacy endpoint included ITT, per protocol population (PPS), observed data analysis and multiple imputation analysis. For all key secondary efficacy endpoints, sensitivity analysis also included PPS population. As pre-specified in the SAP, subgroups of patients with or without neuropathic pain components were analyzed. All analyses were performed using SAS EG 8.2 (SAS 9.4).

Safety data were analyzed in all subjects who were exposed to study treatments, regardless of the amount or duration of treatment. TEAEs were summarized by primary System Organ Class (SOC) and Preferred Term (PT) for each treatment group.

## 3 Results

### 3.1 Subject disposition and baseline characteristics

**Fig. 1:**
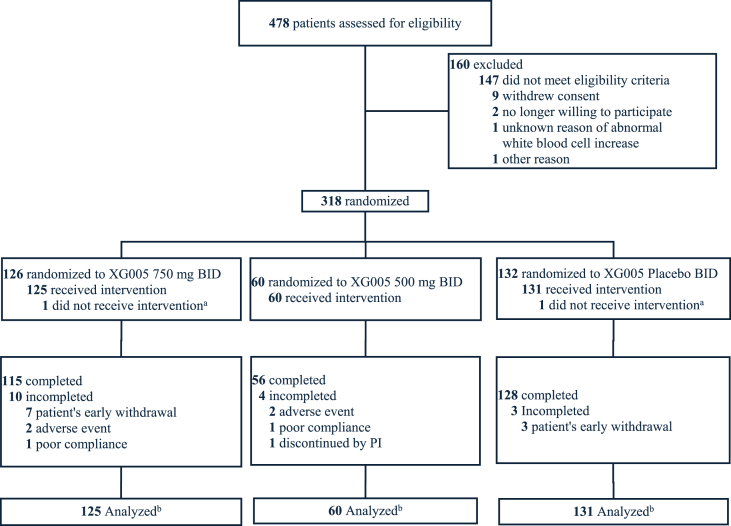
CONSORT flowchart. Notes: ^a^ One randomized patient did not meet eligibility criteria and dropped off immediately after randomization; and one patient withdrew study voluntarily right after randomization, therefore both patients did not receive the study drug. ^b^ Efficacy and safety analysis sets were identical and included all patients who received at least 1 dose of study drug.

The study was conducted at 23 centers in China from November 2022 to May 2024. Fig. 1 summarizes the participant flow. A total of 478 subjects were screened and 318 were randomized and 316 were administered with either XG005 750 mg (n = 125) BID, XG005 500 mg (n = 60), or placebo (n = 131). Overall, the baseline characteristics in age, gender, Body Mass Index (BMI), mental health, quality of life, and baseline knee pain, function and KL grading were balanced across the groups, with no significant differences. Among the 316 treated subjects, 79.7 % were female and 20.3 % were male; 94.9 % were Han race; subjects with bilateral knee OA accounted 90.8 % and 12.0 % subjects had neuropathic pain components likely (i.e., painDETECT questionnaire score >18) (Table 1).

**Table 1:** Demographics and baseline characteristics of patients.

Characteristics	XG005		Placebo (n = 131)	P values
	750 mg (n = 125)	500 mg (n = 60)		
Age, Mean (SD), y	58.2 (6.8)	60.3 (5.8)	58.7 (6.8)	>0.05
Gender, n (%)				
Male	25 (20.0)	12 (20.0)	27 (20.6)	>0.05
Female	100 (80.0)	48 (80.0)	104 (79.4)	>0.05
Race, n (%)				
Han	115 (92.0)	56 (93.3)	129 (98.5)	>0.05
Others	10 (8.0)	4 (6.7)	2 (1.5)	>0.05
BMI, Mean (SD), kg/m^2^	26.2 (3.5)	26.2 (3.5)	26.0 (2.7)	>0.05
Weekly Average of DWP, Mean (SD), 0–10 NRS	5.9 (0.9)	5.7 (0.7)	5.8 (0.7)	>0.05
painDETECT Score,n (%)				
<13	68 (54.4)	39 (65.0)	75 (57.3)	>0.05
13-18	38 (30.4)	16 (26.7)	42 (32.1)	>0.05
>18	19 (15.2)	5 (8.3)	14 (10.7)	>0.05
KL Grade,n (%)				
II	78 (62.4)	40 (66.7)	83 (63.4)	>0.05
III	47 (37.6)	20 (33.3)	48 (36.6)	>0.05
Unilateral/bilateral knee OA, n	12/113	6/54	11/120	>0.05
WOMAC, Mean (SD), 0–10 NRS				
Total	4.4 (1.5)	4.4 (1.2)	4.4 (1.5)	>0.05
Pain	4.6 (1.3)	4.5 (1.1)	4.6 (1.3)	>0.05
Function	4.5 (1.7)	4.6 (1.4)	4.5 (1.6)	>0.05
Stiffness	3.3 (2.1)	3.4 (2.1)	3.5 (2.2)	>0.05
KOOS, Mean (SD)				
Total	52.9 (14.8)	51.8 (12.7)	53.2 (13.4)	>0.05
Symptoms	59.5 (19.1)	58.5 (19.5)	59.5 (19.1)	>0.05
Stiffness	66.5 (20.2)	65.6 (20.3)	63.8 (20.0)	>0.05
Pain	53.8 (13.8)	52.5 (12.4)	53.0 (12.5)	>0.05
Function, Daily Living	56.2 (16.6)	55.7 (13.9)	57.8 (14.4)	>0.05
Function, Sports and Recreational Activities	34.8 (18.4)	32.9 (17.5)	33.8 (18.6)	>0.05
Quality of Life	44.4 (15.8)	41.5 (13.6)	45.0 (16.0)	>0.05
SF-12, Mean (SD)				
General Health	42.2 (8.6)	41.8 (8.5)	41.8 (8.9)	>0.05
Mental Health	53.1 (7.2)	52.4 (7.3)	51.1 (7.7)	>0.05
Weekly Average of Daily SIS, Mean (SD), 0–10 NRS	2.4 (2.3)	2.2 (2.1)	2.3 (2.2)	>0.05
PHQ-9, Mean (SD)	2.3 (2.8)	2.1 (2.5)	2.2 (2.5)	>0.05
GAD-7, Mean (SD)	1.4 (2.4)	1.1 (1.9)	1.4 (2.3)	>0.05

Abbreviations: BMI, Body Mass Index; DWP, Daily Walking Pain; GAD-7, General Anxiety Disorder; KL, Kellgren-Lawrence; KOOS, Knee Injury and Osteoarthritis Outcome Score; NRS, Numerical Rating Scale; PHQ-9, Patient Health Questionnaire-9; SF-12, 12-Item Short-Form Health Survey; WOMAC, Western Ontario and McMasters Universities Osteoarthritis Index 3.1. P values were comparison across three groups; nominal data were compared with percentage of each group.

### 3.2 Primary, key secondary and other efficacy endpoints

**Fig. 2:**
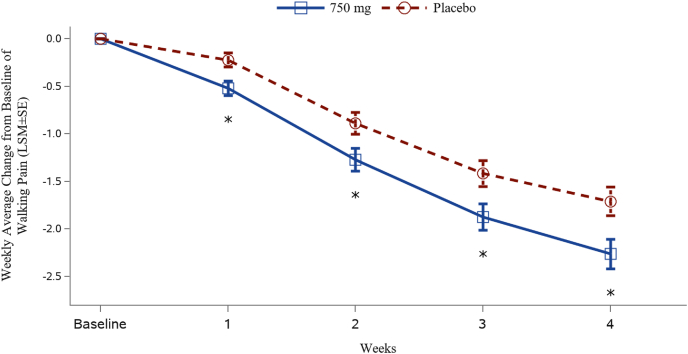
Change from baseline in weekly average of daily walking pain over time. Notes: The least-squares means (LSM±SE) and *P*-values are obtained from analysis of covariance (ANCOVA) model with treatment and sex as categorical fixed effects, and baseline walking pain score as a covariate. ∗*P* = 0.0025, 0.0113, 0.0096 and 0.0055 for 750 mg XG005 versus placebo at Weeks 1, 2, 3 and 4, respectively.

As the primary efficacy endpoint, the least squares mean (LSM) (95 % CI) of change from baseline in WADWP at Week 4 was −2.26 (−2.57, −1.95) for the 750 mg group and −1.71 (−2.01, −1.41) for the placebo group. The LSM difference (95 % CI) between the 750 mg group and the placebo group was −0.55 (−0.94, −0.16) and *P* = 0.0055. Statistically significant improvement in WADWP was observed since week 1 and maintained for the entire study period (Fig. 2).

The robustness of the primary endpoint result was confirmed by sensitivity analyses using the per protocol set (PPS), observed data, and multiple imputations. The LSM difference (95 % CI) between the 750 mg group and the placebo group was −0.74 (−1.14, −0.34) and *P* = 0.0003; −0.72 (−1.10, −0.34) and *P* = 0.0002; and −0.63 (−1.01, −0.25) and *P* = 0.0012 using PPS, observed data and multiple imputations, respectively.

In terms of change from baseline in WADWP measure, XG005 at 500 mg did not show statistical difference from the placebo. 750 mg XG005 had numerically greater improvement than 500 mg XG005 from week 1–4 and was statistically different at week 3 (*P* = 0.044).

**Fig. 3:**
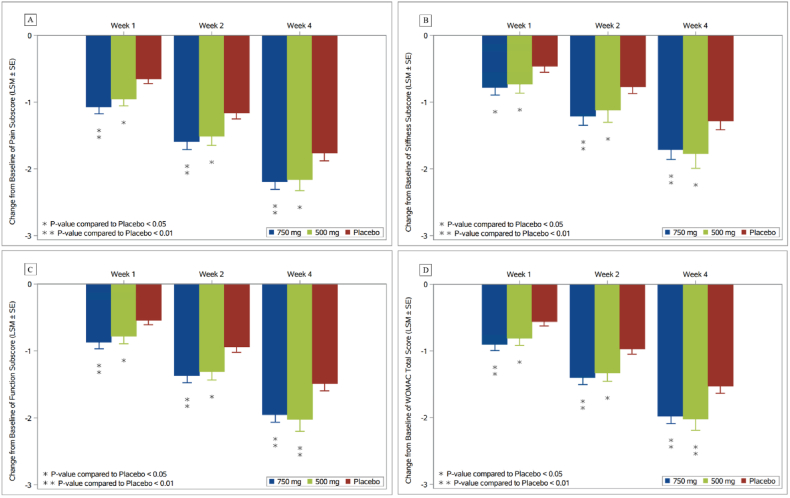
Change from baseline in WOMAC subscores and total score over time. Abbreviations: LSM, Least-squares mean; SE, Standard error; WOMAC, Western Ontario and McMasters Universities Osteoarthritis Index. The LS means (±SE) and *P*-values are obtained from Analysis of covariance (ANCOVA) models with treatment and sex as categorical fixed effects, and corresponding baseline WOMAC subscore as a covariate. All measures are based on 0–10 numerical rating scale.

As the key secondary efficacy endpoints, compared with the placebo group, the LSM difference (95 % CI) in the change from baseline of the WOMAC pain at week 4 was −0.43 (−0.74, −0.13) with *P* = 0.0056 for the 750 mg group, and −0.40 (−0.78, −0.02) with *P* = 0.0384 for the 500 mg group. Fig. 3 summarizes the WOMAC pain, stiffness, function and total score over time for both XG005 treatment and placebo groups. For each XG005 dose, the difference over placebo was statistically significant at each time point. The high dose XG005 showed numerical advantage over the low dose XG005.

With respect to the KOOS pain subscale, compared with the placebo group, the LSM difference (95 % CI) in the change from baseline in the KOOS pain subscale score of the study knee at week 4 was 5.56 (2.69, 8.42) with *P* = 0.0002 for the 750 mg group, and 3.74 (0.17–7.31) with *P* = 0.0402 for the 500 mg group. The change from baseline for KOOS total score was statistically significantly greater in both XG005 groups over placebo (*P* < 0.05, Table 2). All KOOS sub domain scores (i.e., knee pain, stiffness, symptoms, function and daily living, sports and recreational activities, knee-related QoL) showed statistically improvement in favor of XG005 (*P* < 0.05, Table 2). There was no statistical difference between high and low dose of XG005 in KOOS measures.

**Table 2:** Change from baseline in Knee Injury and Osteoarthritis Outcome Score (KOOS).

Subscore	750 mg XG005 vs Placebo (n = 125/131)		500 mg XG005 vs Placebo (n = 60/131)	
Visit	LSM Difference (95 % CI)	*P*-value	LSM Difference (95 % CI)	*P*-value
**KOOS Pain**				
Week 1	2.44 (0.53, 4.35)	0.0125	2.13 (−0.26, 4.51)	0.0801
Week 2	4.46 (1.99, 6.93)	0.0004	2.67 (−0.42, 5.75)	0.0897
Week 4	5.56 (2.69, 8.42)	0.0002	3.74 (0.17, 7.31)	0.0402
**KOOS Symptom**				
Week 1	3.21 (1.03, 5.38)	0.0041	2.76 (0.04, 5.48)	0.0464
Week 2	3.86 (1.04, 6.68)	0.0074	5.36 (1.85, 8.87)	0.0029
Week 4	3.94 (0.85, 7.03)	0.0126	3.38 (−0.48, 7.23)	0.0858
**KOOS Knee Stiffness**				
Week 1	2.11 (−0.30, 4.52)	0.0864	2.56 (−0.44, 5.57)	0.0945
Week 2	3.70 (0.84, 6.56)	0.0114	3.23 (−0.33, 6.79)	0.0752
Week 4	4.43 (1.38, 7.47)	0.0045	3.68 (−0.11, 7.48)	0.0572
**KOOS Function, Daily Living**				
Week 1	3.21 (1.08, 5.34)	0.0032	2.43 (−0.22, 5.08)	0.0727
Week 2	4.29 (1.86, 6.72)	0.0006	4.14 (1.12, 7.17)	0.0074
Week 4	3.86 (1.06, 6.65)	0.0071	4.63 (1.14, 8.12)	0.0094
**KOOS Function, Sports and Recreational Activities**				
Week 1	2.61 (−0.15, 5.36)	0.0635	3.14 (−0.29, 6.57)	0.0727
Week 2	5.83 (2.28, 9.38)	0.0014	4.78 (0.35, 9.21)	0.0344
Week 4	3.86 (−0.20, 7.93)	0.0623	4.61 (−0.46, 9.67)	0.0745
**KOOS Knee-related Quality of Life**				
Week 1	1.56 (−0.57, 3.69)	0.1514	1.39 (−1.28, 4.06)	0.3060
Week 2	4.00 (1.28, 6.73)	0.0042	4.03 (0.62, 7.45)	0.0207
Week 4	3.79 (0.46, 7.12)	0.0259	4.34 (0.17, 8.51)	0.0413
**KOOS Total Score**				
Week 1	2.78 (0.98, 4.58)	0.0026	2.45 (0.20, 4.69)	0.0327
Week 2	4.44 (2.17, 6.70)	0.0001	4.07 (1.25, 6.90)	0.0048
Week 4	4.30 (1.66, 6.94)	0.0015	4.31 (1.02, 7.60)	0.0104

Note: Missing scores are imputed using the average value of the data available at the same visit in the same treatment group. LS means, LS mean difference, 95 % CIs and p-values are obtained from Analysis of covariance (ANCOVA) model with treatment and sex as categorical fixed effects, and baseline KOOS subscore as a covariate. P values are comparison with placebo.

### 3.3 PGIC, rescue medication use, sleep interference and SF-12 assessments

Compared with the placebo group, both XG005 treatment groups showed statistically significant improvements in PGIC scores at all time points. The LSM differences (95 % CI) for 750 mg XG005 over placebo at weeks 1, 2, and 4 were −0.35 (−0.51, −0.18) with *P* < 0.0001, −0.32 (−0.50, −0.14) with *P* = 0.0006, and −0.31 (−0.51, −0.10) with *P* = 0.0032, respectively; and for 500 mg over placebo, those were −0.26 (−0.46, −0.05) with *P* = 0.0129, −0.28 (−0.51, −0.06) with *P* = 0.0132, and −0.30 (−0.55, −0.04) with *P* = 0.022, respectively. Greater proportions of subjects rated “very much improved” and “much improved” in 750 mg and 500 mg XG005 groups than in the placebo (*P* = 0.0043 and 0.0193, respectively).

The total amount of acetaminophen consumed per subject over 4 weeks (mean ± SD) was 2248.0 ± 5135.1, 2075.0 ± 4432.0 or 2419.8 ± 5443.7 mg for the high or low dose XG005 or placebo groups, respectively (*P* > 0.05). There was no difference in proportion of subjects who used rescue medication or days of using rescue medication across groups.

Though sleep was only mildly affected at baseline for this study population, the LSM differences (95 % CI) in sleep interference score for 750 mg XG005 over placebo at weeks 3 and 4 were −0.31 (−0.54, −0.07) with *P* = 0.0105 and −0.44 (−0.69, −0.20) with *P* = 0.0004. No difference in SIS between 500 mg XG005 and placebo groups at any timepoints was observed.

Analysis of the SF-12 scores at week 4 showed that the LSM difference (95 % CI) for the 500 mg group over placebo in the "general health" and "mental health" dimensions were 2.40 (0.02, 4.78), *P* = 0.0483 and 2.25 (0.39, 4.12), *P* = 0.0181, respectively, while no statistically significant differences were observed in other dimensions or dose group.

### 3.4 Subgroup analysis of nociceptive pain and neuropathic pain responses

Neuropathic pain components were assessed with painDETECT questionnaires at baseline. Though a small portion of patients had neuropathic pain in their knees, compared to placebo the 750 mg and pooled 750 and 500 mg XG005 groups still improved significantly in KOOS pain and total scores and WOMAC function (*P* < 0.05, Table 3). The LSM differences between XG005 groups and placebo were 2.3-, 3.3- and 3.0-fold more in KOOS pain, KOOS total and WOMAC function, and increased 82.7 %, 41.5 % and 61.2 % in WOMAC pain and stiffness and WADWP respectively for the pooled neuropathic pain subgroup, compared with the pooled nociceptive pain subgroup (Table 3).

**Table 3:** Nociceptive versus neuropathic pain subgroup analysis at week 4.

	Baseline painDETECT score	<13 (neuropathic pain component unlikely)			>18 (neuropathic pain component likely)			*P*-value between LSMDs
		N	LSM Difference (95 % CI)	*P*-value	N	LSM Difference (95 % CI)	*P*-value	
		Active/PBO			Active/PBO			
WADWP	750 mg	68/75	−0.60 (−1.12, −0.08)	0.0241	19/14	−0.75 (−2.03, 0.52)	0.2381	0.9374
	500 mg	39/75	−0.31 (−0.92, 0.31)	0.3282	5/14	−0.92 (−2.71, 0.88)	0.3056	0.5305
	750 + 500 mg pooled	107/75	−0.49 (−0.96, −0.02)	0.0393	24/14	−0.79 (−1.97, 0.38)	0.1799	0.8303
WOMAC pain	750 mg	68/75	−0.56 (−0.93, −0.19)	0.0032	19/14	−0.92 (−2.14, 0.31)	0.1386	0.9955
	500 mg	39/75	−0.44 (−0.88, −0.00)	0.0476	5/14	−1.04 (−2.74, 0.65)	0.2175	0.5857
	750 + 500 mg pooled	107/75	−0.52 (−0.85, −0.18)	0.0025	24/14	−0.95 (−2.09, 0.20)	0.1027	0.8295
WOMAC function	750 mg	68/75	−0.42 (−0.78, −0.07)	0.0204	19/14	−1.06 (−2.24,0.12)	0.0764	0.2431
	500 mg	39/75	−0.43 (−0.85, −0.01)	0.0467	5/14	−1.87 (−3.52, −0.23)	0.0269	0.0277
	750 + 500 mg pooled	107/75	−0.42 (−0.74, −0.10)	0.0095	24/14	−1.27 (−2.37, −0.16)	0.0261	0.0933
WOMAC stiffness	750 mg	68/75	−0.54 (−0.92, −0.16)	0.0055	19/14	−0.62 (−1.98, 0.74)	0.3618	0.8149
	500 mg	39/75	−0.52 (−0.97, −0.07)	0.0230	5/14	−1.07 (−2.92, 0.77)	0.2441	0.4386
	750 + 500 mg pooled	107/75	−0.53 (−0.87, −0.19)	0.0023	24/14	−0.75 (−1.99, 0.50)	0.2326	0.9521
KOOS pain	750 mg	68/75	5.80 (2.34, 9.25)	0.0011	19/14	12.44 (2.42, 22.47)	0.0165	0.1461
	500 mg	39/75	4.55 (0.48, 8.63)	0.0288	5/14	11.17 (−2.81, 25.16)	0.1136	0.2834
	750 + 500 mg pooled	107/75	5.34 (2.25, 8.43)	0.0008	24/14	12.12 (2.88, 21.36)	0.0117	0.1070
KOOS total	750 mg	68/75	4.15 (1.04, 7.26)	0.0092	19/14	12.28 (2.84, 21.72)	0.0123	0.0510
	500 mg	39/75	3.83 (0.15, 7.51)	0.0414	5/14	15.91 (2.61, 29.21)	0.0205	0.0315
	750 + 500 mg pooled	107/75	4.03 (1.25, 6.82)	0.0047	24/14	13.18 (4.44, 21.93)	0.0043	0.0196

Note: Analyses were based on mITT population. P values were comparison with placebo. P values for the comparison of LSMDs are in the last column. WADWP: weekly average of daily walking pain.

### 3.5 Adverse events

The incidence of treatment-emergent adverse events (TEAEs) was 75.2 % (94/125) in the 750 mg group, 65.0 % (39/60) in the 500 mg group, and 38.9 % (51/131) in the placebo group. The incidence of TEAEs related to the study drug was 68.8 % (86/125) in the 750 mg group, 61.7 % (37/60) in the 500 mg group, and 29.8 % (39/131) in the placebo group. Among which, the gastrointestinal (GI) TEAEs were 18.4 % (23/125), 16.7 % (10/60) and 6.1 % (8/131) in the 750 mg, 500 mg and placebo groups, respectively. These drug-related TEAEs were mostly Grade 1 in severity and mainly occurred in the first week of the trial. There were no treatment-related severe or serious AEs. TEAEs leading to early withdrawal were 1.6 % (2/125), 1.7 % (1/60), and 0 % (0/131) in the 750 mg, 500 mg and the placebo groups, respectively.

The most common TEAEs were dizziness, somnolence, nausea and vomiting, reported more in XG005 groups than in the placebo group (Table 4).

**Table 4:** Summary of most common treatment-emergent adverse events (TEAEs).

	XG005		Placebo (n = 131)
	750 mg (n = 125)	500 mg (n = 60)	
**Most Common TEAEs**^a^, n (%)			
Dizziness	65 (52.0)	28 (46.7)	21 (16.0)
Somnolence	30 (24.0)	16 (26.7)	9 (6.9)
Nausea	8 (6.4)	3 (5.0)	0
Vomiting	8 (6.4)	1 (1.7)	0
Upper respiratory tract infection	5 (4.0)	2 (3.3)	6 (4.6)
Lethargy	5 (4.0)	1 (1.7)	0
Dyspepsia	5 (4.0)	0	1 (0.8)
Headache	2 (1.6)	1 (1.7)	5 (3.8)
Asthenia	4 (3.2)	3 (5.0)	2 (1.5)
Weight increased	4 (3.2)	0	0
Abdominal discomfort	3 (2.4)	2 (3.3)	6 (4.6)
Alanine aminotransferase increased	1 (0.8)	2 (3.3)	0
Red blood cells urine	1 (0.8)	2 (3.3)	0
Urinary tract infection	1 (0.8)	3 (5.0)	3 (2.3)
Blurred vision	1 (0.8)	2 (3.3)	0
Upper abdominal pain	0	3 (5.0)	0
Tinnitus	0	2 (3.3)	0

a Occurred in ≥3 % of patients in any treatment group during the treatment period.

## 4 Discussion

In this study, XG005 treatment demonstrated statistically significant analgesic effect and improvement in OA stiffness and function, as well as sleep quality and quality of life. The result for the primary endpoint was supported and confirmed by the key secondary and other efficacy measures and sensitivity analyses. Successful management of knee OA must address both pain and limitation of mobility, whose interactions remain poorly understood [19]. Stiffness and dysfunction affect patient's overall assessment of heath, quality of life and mental health [20]. So far, few approved analgesics have stiffness and function improvement included in their product labeling. The study results encourage further testing of OA symptoms (i.e., pain, stiffness and function) as co-primary efficacy endpoints in future trials, to earn symptom modification on the label in addition to pain amelioration.

OA pain is a complex pathophysiology, including local inflammation involving all joint structures, peripheral and central neuropathic abnormalities [7,21]. Approximately 50 % of OA patients present with clinical neuropathic pain, often strongly associated with long duration of severe pain, limitation of mobility, sleep, social and emotional dysfunction [[22], [23], [24]]. Neuropathic pain in OA is resistant to anti-inflammatory therapeutics and therapeutics targeting neuropathic pain is needed [7]. In this trial, neuropathic pain was assessed with validated painDETECT questionnaires. XG005 effects on KOOS pain and total and WOMAC function in those patients were 2-3-fold stronger compared to patients with nociceptive pain only. Given the small proportion of neuropathic pain patients enrolled, and the comparison was measured as subgroup analysis (neither as the primary nor as key secondary efficacy endpoints), the above observed trend was far from conclusive in this pilot study. To confirm, future studies need to cap comparable numbers of patients enrolled into neuropathic pain group and non-neuropathic pain group, powered sufficiently and analyzed as the primary or key secondary endpoint. There are no approved therapeutics for OA neuropathic pain yet [25], and integrated multimodal approaches have been recommended [26]. The dual action of XG005 offers a potential opportunity to treat neuropathic pain components beyond suppressing inflammation in OA patients once confirmed.

The daily walking pain, WOMAC pain and KOOS pain improved from baseline by 38.4 %, 47.0 % and 32.6 % at Week 4 in the 750 mg treatment group, which exceeded minimum clinically important improvement [27]. The effect sizes over placebo for the primary efficacy endpoint (daily walking pain) and key secondary efficacy endpoints (WOMAC pain and KOOS pain) at week 4 were 0.36, 0.33, and 0.45, respectively. These effect sizes were small (i.e., within the range of 0.2–0.5) [27]. Though numerically greater LSMDs over placebo were observed with primary efficacy endpoint and WOMAC pain in subjects with neuropathic pain than with nociceptive pain, their effect sizes were barely changed due to small sample size and large standard deviation. However, the effect size of KOOS pain reached 1.05 in neuropathic pain subpopulation, reflecting a large effect (i.e., >0.8) [27].

By conjugating naproxen and pregabalin, XG005 synchronizes the T_max_ of pregabalin and naproxen and elevates their C_trough_ levels by approximately 73 % and 27 %, respectively, compared to bioequivalent doses of pregabalin and naproxen taken as a fixed-dose combination (data from phase 1 trials). 750 mg XG005 releases bioequivalent doses of 300 mg naproxen and 150 mg pregabalin, and 500 mg XG005 releases 200 mg of naproxen and 120 mg pregabalin (data on file). Those doses are well below the commonly used dose of naproxen 500 mg BID for OA pain [28,29], 300 mg BID for pregabalin in chronic neuropathic pain [30] and 150 mg pregabalin BID being tested in a placebo-controlled pilot hand OA trial [31]. Nevertheless, XG005 demonstrated improved efficacy (particularly in the neuropathic pain subgroup) than naproxen or other NSAIDs used at much higher doses for OA [17,28,29], which remains to be confirmed in additional trials. Interestingly, analgesic effects were also observed with 250 mg XG005 (containing 120 mg naproxen and 65 mg pregabalin) on day 2 of the dose titration phase (the LSM difference (95 % CI) in change from baseline of daily walking pain between the XG005 and the placebo groups was −0.18 (−0.35, −0.00) and *P* = 0.0467). The potency and early onset effect could accommodate individualized treatment plans based on therapeutic need and patient response. Naproxen blocks prostaglandin production, reducing inflammation and inflammatory stimuli on nociceptive nerve [32], while pregabalin decreases the hyperexcitability of peripheral and central neurons caused by damaged nerve tissue or stimulation [33]. Hence, dual-acting XG005 may represent a potential advantage in treating OA pain if a synergy could be established by including proper comparison treatments in future trials.

XG005 was generally safe and well tolerated with a safety profile consistent with the tolerability profiles described in the approved package inserts for naproxen and pregabalin. No new safety signals were identified. Since XG005 is not active inside gastrointestinal (GI) tract until being cleaved into active drugs after absorption, GI mucosa does not have the direct exposure to local high dose of naproxen *per se*, thus, potentially avoids direct cytotoxicity (e.g., membrane permeabilization, necrosis and apoptosis of GI mucosal cells) independent of the inhibition of COX activity [34,35]. Pre-clinical animal studies demonstrated GI advantage of XG005 compared with ingestion of dose-comparable naproxen (data on file). This trial showed that GI side effects occurred in 18.4 % and 16.7 % subjects in 750 mg and 500 mg groups, respectively. Those side effects were mild in severity, and none led to study discontinuation. In knee and/or hip OA trial with naproxen, the incidence of GI AEs was 35 % and 8 % of subjects discontinued the study due to GI AEs [28,29,36]. Thus, XG005 may increase the GI tolerance for chronic use. Given analgesic effects observed with 250 mg XG005, this would further reduce GI risk.

The limitation of the study includes short treatment and follow-up period. Prolonged treatment to be studied may result in improved efficacy as an abatement of neuroplasticity and central sensitization takes time after eliminating pain input from the joint [37]. Also, longer observation allows better characterization of safety profiles.

Compared with 500 mg XG005, 750 mg XG005 showed statistically greater improvement in daily walking pain and some numerical advantages in WOMAC, KOOS pain, PGIC and SIS measures. In terms of safety, slightly more patients reported dizziness, vomiting and dyspepsia in 750 mg XG005 group than in the 500 mg XG005 group.

To summarize, XG005 as a novel, non-opioid new chemical molecule acting on both inflammatory pain and neuropathic pain pathways, demonstrated robust efficacy on modifying symptoms of OA with favorable GI safety profile. Its effect on treating OA with neuropathic pain is particularly encouraging for further studies to confirm.

## Authors contribution

All mentioned authors have made substantial contributions to the conception or design of the work; or the acquisition, analysis, or interpretation of data for the work; helped drafting the work or reviewing it critically for important intellectual content; gave final approval of the version to be published; and agreed to be accountable for all aspects of the work in ensuring that questions related to the accuracy or integrity of any part of the work are appropriately investigated and resolved.

Corresponding author GLJ takes responsibility for the integrity of the complete work, from inception to finished manuscript.

## Role of the funding source

This study was designed, analyzed, interpreted, reported and funded by Xgene Pharmaceutical, Inc.

## Conflict of interest statement

GLJ is an employee of Xgene Pharmaceutical, Inc. and he owns stocks of the company. The rest of the authors were principal investigators of the trial and were sponsored with research grants from Xgene Pharmaceutical.

## References

[1] Kloppenburg M., Namane M., Cicuttini F. (2025). Osteoarthritis. Lancet.

[2] Hunter D.J., Bierma-Zeinstra S. (2019). Osteoarthritis. Lancet.

[3] Overton C., Nelson A.E., Neogi T. (2022). Osteoarthritis treatment guidelines from six professional societies: similarities and differences. Rheum. Dis. Clin. N. Am..

[4] Arden N.K., Perry T.A., Bannuru R.R., Bruyère O., Cooper C., Haugen I.K., Hochberg M.C., McAlindon T.E., Mobasheri A., Reginster J.Y. (2021). Non-surgical management of knee osteoarthritis: comparison of ESCEO and OARSI 2019 guidelines. Nat. Rev. Rheumatol..

[5] Saxer F., Hollinger A., Bjurström M.F., Conaghan P.G., Neogi T., Schieker M. (2024). Pain-phenotyping in osteoarthritis: current concepts, evidence, and considerations towards a comprehensive framework for assessment and treatment. Osteoarthr. Cartil. Open.

[6] Woolf C.J. (2011). Central sensitization: implications for the diagnosis and treatment of pain. Pain.

[7] Thakur M., Dickenson A.H., Baron R. (2014). Osteoarthritis pain: nociceptive or neuropathic?. Nat. Rev. Rheumatol..

[8] Petersen K.K., O'Neill S., Blichfeldt-Eckhardt M.R., Nim C., Arendt-Nielsen L., Vægter H.B. (2025). Pain profiles and variability in temporal summation of pain and conditioned pain modulation in pain-free individuals and patients with low back pain, osteoarthritis, and fibromyalgia. Eur. J. Pain.

[9] Jiang G.-L., Evanson J.R., Solanki D., D'Aunno D., DeNoia E., Rondon J.C. (2025). Synchronized inhibition of both nociceptive and neuropathic pain signals with XG005 for acute pain (abstract). British pain society 58th annual scientific meeting. Br. J. Pain.

[10] Krupka E., Jiang G.L., Jan C. (2019). Efficacy and safety of intra-articular injection of tropomyosin receptor kinase A inhibitor in painful knee osteoarthritis: a randomized, double-blind and placebo-controlled study. Osteoarthr. Cartil..

[11] Arendt-Nielsen L., Jiang G.L., DeGryse R., Turkel C. (2016). Intra-articular onabotulinumtoxinA in osteoarthritis knee pain: effect on human mechanistic pain biomarkers and clinical pain. Scand. J. Rheumatol..

[12] McAlindon T.E., Driban J.B., Henrotin Y., Hunter D.J., Jiang G.L., Skou S.T. (2015). OARSI clinical trials recommendations: design, conduct, and reporting of clinical trials for knee osteoarthritis. Osteoarthr. Cartil..

[13] Zhang Z., Huang C., Jiang Q., Zheng Y., Liu Y., Liu S. (2020). Guidelines for the diagnosis and treatment of osteoarthritis in China (2019 edition). Ann. Transl. Med..

[14] Ho K.Y., Cardosa M.S., Chaiamnuay S., Hidayat R., Ho H.Q.T., Kamil O. (2020). Practice advisory on the appropriate use of NSAIDs in primary care. J. Pain Res..

[15] Altman R., Asch E., Bloch D., Bole G., Borenstein D., Brandt K. (1986). Development of criteria for the classification and reporting of osteoarthritis. Classification of osteoarthritis of the knee. Diagnostic and therapeutic criteria committee of the American rheumatism association. Arthritis Rheum..

[16] Essex M.N., O'Connell M.A., Behar R., Bao W. (2016). Efficacy and safety of nonsteroidal anti-inflammatory drugs in Asian patients with knee osteoarthritis: summary of a randomized, placebo-controlled study. Int J Rheum Dis.

[17] Bannuru R.R., Schmid C.H., Kent D.M., Vaysbrot E.E., Wong J.B., McAlindon T.E. (2015). Comparative effectiveness of pharmacologic interventions for knee osteoarthritis: a systematic review and network meta-analysis. Ann. Intern. Med..

[18] Jung S.Y., Jang E.J., Nam S.W., Kwon H.H., Im S.G., Kim D. (2018). Comparative effectiveness of oral pharmacologic interventions for knee osteoarthritis: a network meta-analysis. Mod. Rheumatol..

[19] Crow J.A., Fillingim R.B. (2022). Working toward mechanistic pain phenotyping in osteoarthritis. Osteoarthr. Cartil..

[20] Wojcieszek A., Kurowska A., Majda A., Liszka H., Gądek A. (2022). The impact of chronic pain, stiffness and difficulties in performing daily activities on the quality of life of older patients with knee osteoarthritis. Int. J. Environ. Res. Publ. Health.

[21] Perrot S., Anne-Priscille T. (2023). Pain in osteoarthritis from a symptom to a disease. Best Pract. Res. Clin. Rheumatol..

[22] Mougui A., Belouaham S., El Bouchti I. (2023). Neuropathic pain in patients with primary knee osteoarthritis: a cross-sectional study. Rom. J. Intern. Med..

[23] Güngör Demir U., Demir A.N., Toraman N.F. (2021). Neuropathic pain in knee osteoarthritis. Adv. Rheumatol,.

[24] Garip Y., Eser F., Kilicarslan A., Bodur H. (2015). Prevalence of neuropathic pain in rheumatic disorders: association with disease activity, functional status and quality of life. Arch. Rheumatol..

[25] Shinu P., Morsy M.A., Nair A.B., Mouslem A.K.A., Venugopala K.N., Goyal M. (2022). Novel therapies for the treatment of neuropathic pain: potential and pitfalls. J. Clin. Med..

[26] Hange N., Poudel S., Ozair S., Paul T., Nambakkam M., Shrestha R. (2022). Managing chronic neuropathic pain: recent advances and new challenges. Neurol. Res. Int..

[27] Dworkin R.H., Turk D.C., Wyrwich K.W., Beaton D., Cleeland C.S., Farrar J.T. (2008). Interpreting the clinical importance of treatment outcomes in chronic pain clinical trials: IMMPACT recommendations. J. Pain.

[28] Kivitz A.J., Moskowitz R.W., Woods E., Hubbard R.C., Verburg K.M., Lefkowith J.B. (2001). Comparative efficacy and safety of celecoxib and naproxen in the treatment of osteoarthritis of the hip. J. Int. Med. Res..

[29] Lisse J., Espinoza L., Zhao S.Z., Dedhiya S.D., Osterhaus J.T. (2001). Functional status and health-related quality of life of elderly osteoarthritic patients treated with celecoxib. J. Gerontol. A Biol. Sci. Med. Sci..

[30] Dworkin R.H., Corbin A.E., Young J.P., Sharma U., LaMoreaux L., Bockbrader H. (2003). Pregabalin for the treatment of postherpetic neuralgia: a randomized, placebo-controlled trial. Neurology.

[31] Sofat N., Harrison A., Russell M.D., Ayis S., Kiely P.D., Baker E.H. (2017). The effect of pregabalin or duloxetine on arthritis pain: a clinical and mechanistic study in people with hand osteoarthritis. J. Pain Res..

[32] Gilman K.E., Limesand K.H. (2021). The complex role of prostaglandin E_2_-EP receptor signaling in wound healing. Am. J. Physiol. Regul. Integr. Comp. Physiol..

[33] Verma V., Singh N., Singh Jaggi A. (2014). Pregabalin in neuropathic pain: evidences and possible mechanisms. Curr. Neuropharmacol..

[34] Sinha M., Gautam L., Shukla P.K., Kaur P., Sharma S., Singh T.P. (2013). Current perspectives in NSAID-induced gastropathy. Mediat. Inflamm..

[35] Jiang G.L., Im W.B., Donde Y., Wheeler L.A. (2009). EP4 agonist alleviates indomethacin-induced gastric lesions and promotes chronic gastric ulcer healing. World J. Gastroenterol..

[36] Bensen W.G., Fiechtner J.J., McMillen J.I., Zhao W.W., Yu S.S., Woods E.M., Hubbard R.C., Isakson P.C., Verburg K.M., Geis G.S. (1999). Treatment of osteoarthritis with celecoxib, a cyclooxygenase-2 inhibitor: a randomized controlled trial. Mayo Clin. Proc..

[37] Aranda-Villalobos P., Fernández-de-Las-Peñas C., Navarro-Espigares J.L., Hernández-Torres E., Villalobos M., Arendt-Nielsen L. (2013). Normalization of widespread pressure pain hypersensitivity after total hip replacement in patients with hip osteoarthritis is associated with clinical and functional improvements. Arthritis Rheum..

